# Cell cycle control and seed development

**DOI:** 10.3389/fpls.2014.00493

**Published:** 2014-09-23

**Authors:** Ricardo A. Dante, Brian A. Larkins, Paolo A. Sabelli

**Affiliations:** ^1^Embrapa Agricultural InformaticsCampinas, Brazil; ^2^Department of Agronomy and Horticulture, University of NebraskaLincoln, NE, USA; ^3^School of Plant Sciences, University of ArizonaTucson, AZ, USA

**Keywords:** cell division, cotyledon, cyclin-dependent kinase, embryo, endoreduplication, endosperm, retinoblastoma-related, seed coat

## Abstract

Seed development is a complex process that requires coordinated integration of many genetic, metabolic, and physiological pathways and environmental cues. Different cell cycle types, such as asymmetric cell division, acytokinetic mitosis, mitotic cell division, and endoreduplication, frequently occur in sequential yet overlapping manner during the development of the embryo and the endosperm, seed structures that are both products of double fertilization. Asymmetric cell divisions in the embryo generate polarized daughter cells with different cell fates. While nuclear and cell division cycles play a key role in determining final seed cell numbers, endoreduplication is often associated with processes such as cell enlargement and accumulation of storage metabolites that underlie cell differentiation and growth of the different seed compartments. This review focuses on recent advances in our understanding of different cell cycle mechanisms operating during seed development and their impact on the growth, development, and function of seed tissues. Particularly, the roles of core cell cycle regulators, such as cyclin-dependent-kinases and their inhibitors, the Retinoblastoma-Related/E2F pathway and the proteasome-ubiquitin system, are discussed in the contexts of different cell cycle types that characterize seed development. The contributions of nuclear and cellular proliferative cycles and endoreduplication to cereal endosperm development are also discussed.

## INTRODUCTION

### SEED DEVELOPMENT PHASES

Angiosperms reproduce sexually via the production of seeds, which are typically derived from the fertilization of ovules, or asexually via apomixis. Mature seeds characteristically contain three major structures: a sporophyte (the embryo), nutrient storage tissues or organs (the endosperm and/or the embryonic cotyledons), which support embryogenesis and early post-embryonic sporophyte development, and a protective structure (the seed coat or testa). A prominent triploid endosperm is typically present in mature monocot seeds, while most of its cells are consumed during dicot seed development. The seed coat is a maternal structure derived from the ovule integuments that functions in seed protection, dormancy, germination, and, in some dicot species like legumes (Fabaceae or Leguminoseae family), transiently in nutrient storage. In the monocot Poaceae family (grasses), the seed coat is fused to the pericarp (the fruit coat). The maternal plant makes significant contributions to seed production by providing nutrients, conveying hormonal and environmental cues and imposing mechanical constraints on the floral structures within which seeds develop. Consequently, seed production is influenced by a range of zygotic, sporophytic, and environmental factors (Egli, 2006).

The size of a multicellular organism, its organs and tissues depend on the number and size of constituent cells. Cell number, in turn, depends on the rate of cell division, the number of dividing cells, and the duration of the cell proliferation phase during development, while the size of non-dividing cells is influenced by cell growth and cell expansion (defined as increases in cell macromolecular mass and cell volume, respectively; Sugimoto-Shirasu and Roberts, 2003). In plants, cell number generally seems to make a larger contribution than cell size to the size of comparable organs (reviewed by Mizukami, 2001). However, seed size and weight are highly influenced by cell size via the growth and expansion brought about by massive accumulation of storage compounds (proteins, lipids, and/or carbohydrates) and water intake by cotyledon or endosperm cells. In cereal and legume crops, seed growth and development typically comprise three partially overlapping phases: an initial lag phase initiated at fertilization and characterized by cell proliferation and minimal dry weight gain (phase I); seed filling, a linear phase of large dry weight gain associated with cell enlargement and accumulation of storage compounds (phase II); and a final phase of reduced dry weight gain associated with desiccation and dormancy (phase III; Egli, 2006). In phase I, various seed tissues and domains are specified and established, including the vital transfer cells, a filial conduit with the mother plant vascular tissue that nourishes the developing seed. Phase I is also characterized by uptake of sucrose, which is rapidly converted to hexoses via cell wall-bound invertase activity. Even though phase I is critical for seed development and grain yield, its contribution is indirect, as the cells generated in this phase are very small and contribute little to seed biomass. However, during phase II most of the endosperm or cotyledon cells generated in phase I accumulate storage compounds. Thus, phase II is characterized by cell enlargement due to cell growth and cell expansion, and a peak in seed water content (Egli, 2006). In storage cells of the cereal endosperm and legume cotyledons, phase II is also characterized by endoreduplication (also known as endopolyploidization, endocycling, or endoreplication), a type of cell cycle that leads to polyploidy. During phase III, water concentration decreases dramatically and physiological maturity is reached.

### CELL CYCLE TYPES OCCURRING DURING SEED DEVELOPMENT

**FIGURE 1:**
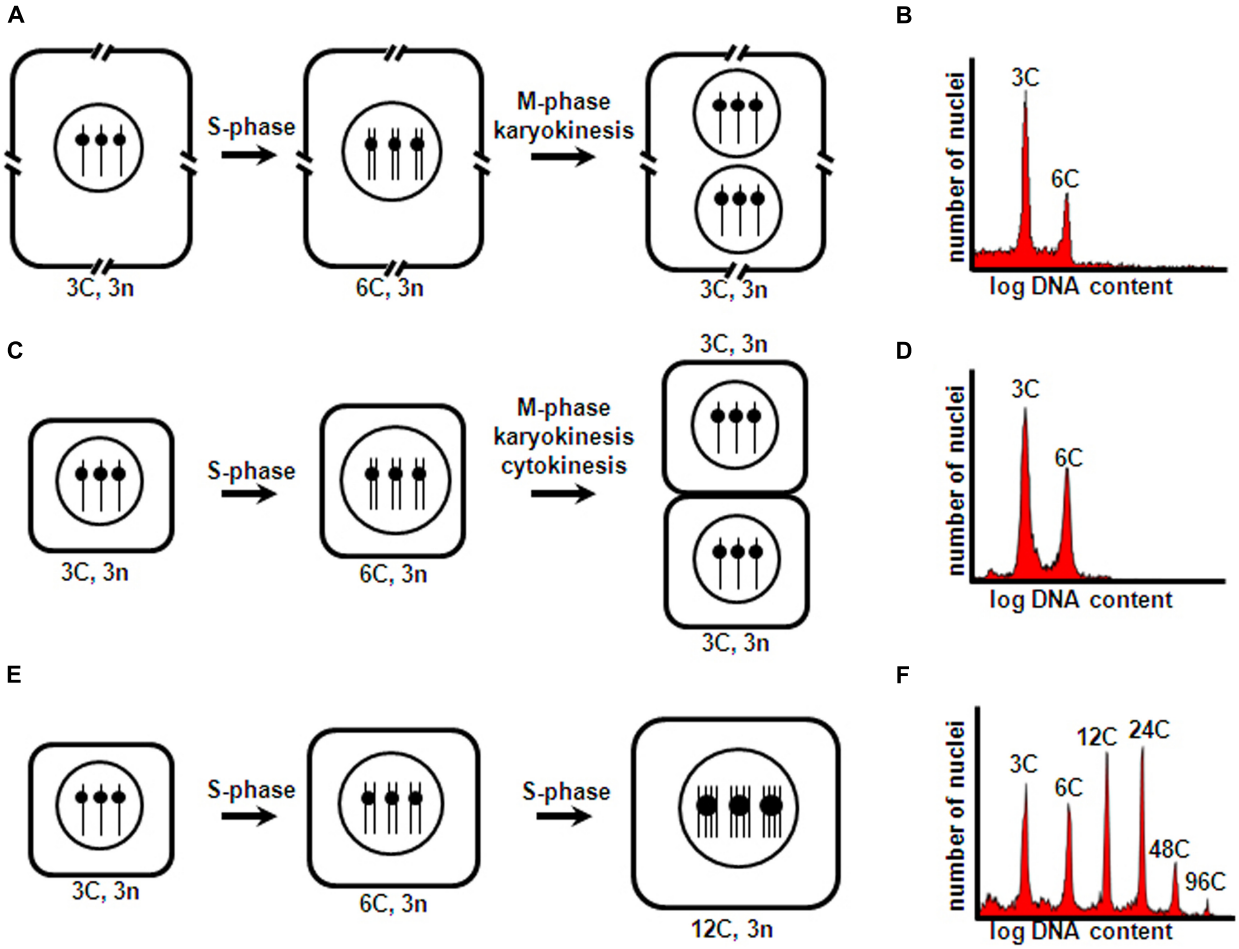
**Cell cycle types occurring during seed development.** Triploid endosperm mother cells (with two maternal and one paternal chromosomal complements) are shown as an example. A hypothetical haploid number *n* = 1 is assumed for simplicity. **(A)** Acytokinetic mitosis of endosperm nuclei within the embryo sac central cell, resulting in a syncytium; **(C)** Cell proliferation through mitotic cell division following syncytium cellularization; **(E)** Endoreduplication of inner endosperm starchy cells. Cell number, size, DNA content, and chromosome number correspond to one complete cell cycle round comprising S-phase and accompanying M-phase and karyokinesis **(A,C)** and cytokinesis **(C)**, and two complete endoreduplication cycle rounds (each comprising S-phase not followed by M-phase, karyokinesis and cytokinesis) **(E)**. Interrupted cell boundaries in **(A)** indicate the large size of the embryo sac central cell. C and n, DNA content and chromosome number of a haploid cell, respectively. **(B,D,F)** show typical nuclear flow-cytometric profiles obtained for tissues undergoing asynchronous, iterative acytokinetic mitosis, mitotic cell division, and endoreduplication cycles, respectively.

Briefly, the prototypical mitotic cycle consists of a DNA replication phase (S-phase), and a chromosome condensation and sister chromatid segregation phase (M-phase), which are preceded by G1 and G2 gap phases, respectively. Typically, this cell cycle is associated with cell division, and M-phase is generally coupled to karyokinesis and cytokinesis, which generate daughter cells with chromosome number and nuclear DNA content identical to those of their mother cell. However, in several plant tissues, cell types, and developmental stages, alternative cell cycle types can occur. In the context of seed development, frequently these are acytokinetic mitosis and endoreduplication (**Figure 1**). In acytokinetic mitosis, the mitotic cell cycle is coupled to karyokinesis in the absence of cytokinesis, thus producing a syncytium or multinucleate cell. Endoreduplication is characterized by recurrent and alternating gap and S-phase, without intervening sister chromatid segregation, karyokinesis and cytokinesis, thus resulting in polyploid cells with an unaltered number of chromosomes, but with each chromosome containing multiple chromatids (reviewed by Edgar et al., 2014). All these different cell cycle types influence the growth and development of seed structures. Early in development, asymmetric cell divisions in the embryo generate polarized daughter cells that take on diverse differentiation paths, and rapid nuclear proliferation and cellularization in the endosperm establish the initial cell populations that occupy the embryo sac. Subsequent intense cell proliferation, coupled to cell differentiation, essentially produces all the embryo and endosperm cell types and tissues. Finally, endoreduplication occurs, which is inherently associated with cell enlargement and accumulation of storage compounds in specialized cotyledon or endosperm cells.

Seed biology aspects such as comparative development and anatomy of seed structures and their underlying signaling networks were reviewed in-depth recently (Sabelli and Larkins, 2009b; Nowack et al., 2010; Lau et al., 2012; Sabelli, 2012b). Likewise, the role of cell cycle regulation in plant growth and development has also been reviewed thoroughly elsewhere (De Veylder et al., 2011; Heyman and De Veylder, 2012; Edgar et al., 2014; Sabelli, 2014). Hence, we focus on recent findings that clarify the role of core cell cycle regulators and different cell cycle types in the development, growth, and function of seed structures.

## CELL CYCLE CONTROL AND CORE REGULATORS IN PLANTS: AN OVERVIEW

### CYCLIN-DEPENDENT KINASES AND CYCLINS

**FIGURE 2:**
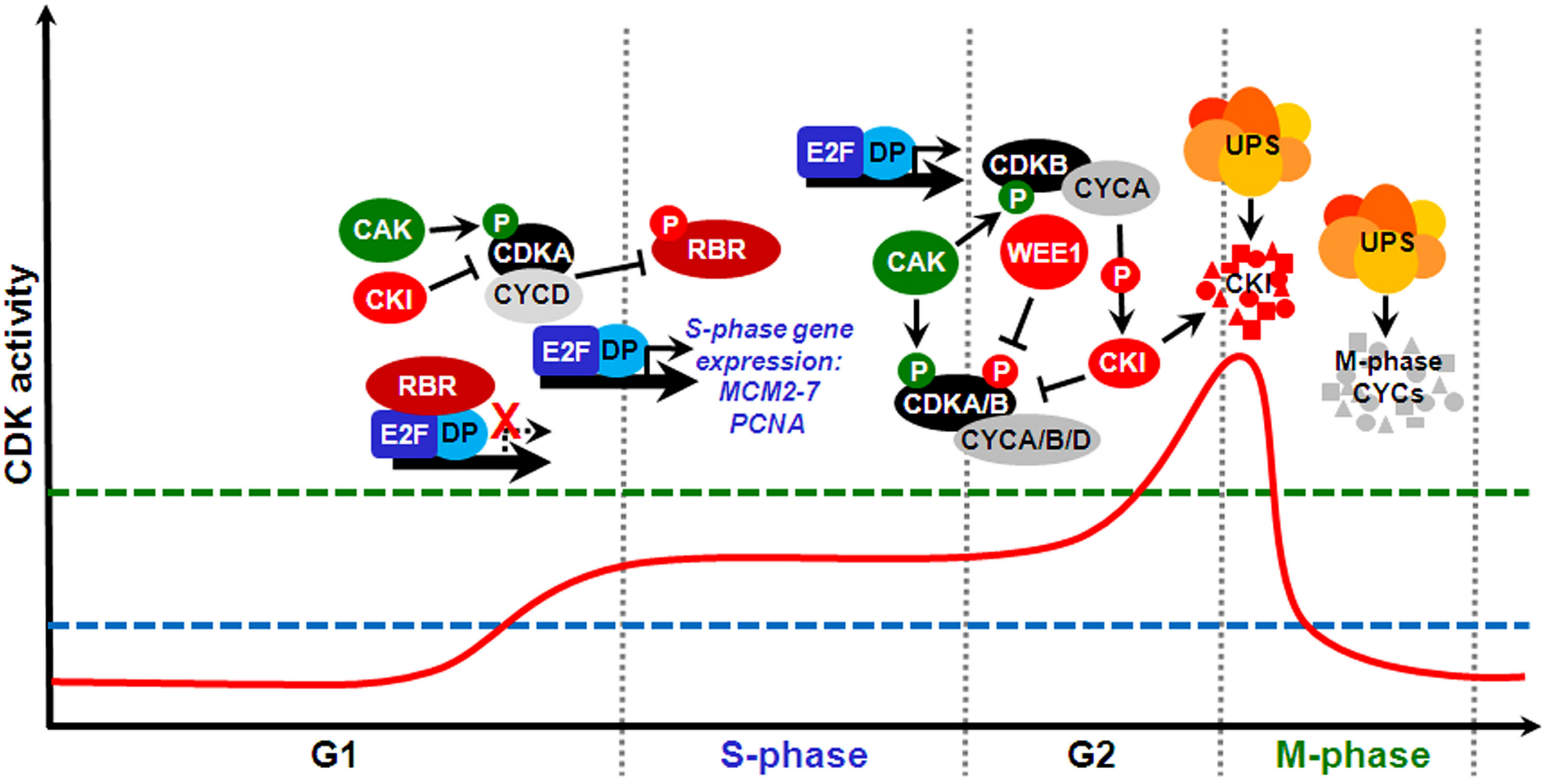
**The prototypical mitotic cell division cycle and some key molecular mechanisms that regulate its major transitions in plants.** Cell cycle progression through distinct phases is driven by periodic fluctuations of cyclin-dependent kinase (CDK) activity (solid red line). Green and red circles labeled with a P letter show stimulatory and inhibitory phosphorylation, respectively. For cells to make a transition from G1 into S-phase and execute DNA replication, CDK activity must surpass an S-phase threshold (dashed blue line). A further CDK activity increase above an M-phase threshold (dashed green line) during G2 drives entry into mitosis. Both M-phase exit and origin of replication licensing require CDK activity to be reduced at the end of mitosis and maintained at low levels for most of G1. At the G1/S transition, CDKA/CYCD complexes phosphorylate and thus inactivate retinoblastoma-related (RBR), thus permitting heterodimeric E2F/DP transcription factors to stimulate an S-phase gene expression program, which results in the expression of MCM2-7 and proliferating cell nuclear antigen (PCNA) genes, among many others. CDKA/CYCD activity is positively and negatively regulated by, respectively, CDK-activating kinase (CAK)-dependent phosphorylation and binding by CKIs. Later in G2, CDK activity requires association with mitotic cyclins and is also stimulated by CAK and inhibited by CKIs. In addition, this CDK activity is inhibited by phosphorylation at specific tyrosine residues by WEE1. Certain CDKs of the B-type, whose expression is E2F/DP-dependent, promote M-phase progression by mechanisms that include interaction with A-type cyclins and stimulation of downstream CDK activity by phosphorylating and targeting certain CKIs for proteolysis by the ubiquitin-proteasome system (UPS) via different E3 ubiquitin ligases. Conversely, mitotic cyclin proteolysis by the UPS, via the APC/C, causes CDK activity to decline sharply, which is required for M-phase exit.

In eukaryotes, cell cycle progression is controlled by the periodic activity of various heterodimeric threonine/serine protein kinases composed of catalytic and regulatory subunits, a cyclin-dependent kinase (CDK) and a cyclin, respectively. Plants possess relatively large sets of genes encoding different CDKs and cyclins, which can interact to form a potentially large number of combinations (Van Leene et al., 2011). Plants contain eight types of CDK-like proteins (reviewed by Dudits et al., 2007). Among the major CDKs involved with cell cycle regulation are members of the A-type, which characteristically contain in their cyclin-interacting α-helix a hallmark PSTAIRE amino acid motif; these function during S-phase and at the G1/S and G2/M transitions. In the plant-specific B-type CDKs, which function primarily at the G2/M transition, the PSTAIRE motif is replaced by PPTALRE (B1-subtype) or PPTTLRE (B2-subtype). D- and F-type CDKs, also known as CDK-activating kinases (CAKs), regulate A- and B-type CDKs through phosphorylation of specific residues (reviewed by Inzé and De Veylder, 2006). Angiosperm genomes possess a cyclin complement of ∼50–60 genes organized into ∼10 types (Wang et al., 2004; La et al., 2006; Hu et al., 2010; Ma et al., 2013). The majority of D-type cyclins are involved in the control of the G1/S transition; A-type cyclins, S-phase, and the G2/M transition; and B-type cyclins, G2/M, and intra-mitotic transitions (Inzé and De Veylder, 2006). CDK/cyclin complexes are subjected to different levels of regulation, including binding by non-catalytic CDK-specific inhibitors (CKIs), activating or inhibitory phosphorylation of CDK subunits, and cell cycle phase-specific cyclin synthesis and proteolysis, the latter of which is mediated by the ubiquitin-proteasome system (UPS; Inzé and De Veylder, 2006). A simplified diagram depicting some major molecular mechanisms of the plant cell cycle is shown in **Figure 2**.

### THE RETINOBLASTOMA-RELATED PATHWAY

In higher eukaryotes, proteins of the retinoblastoma-related (RBR) family are known as repressors of the G1/S transition for their inhibitory effect on heterodimeric E2F/DP transcription factors. These, in turn, control the expression of multiple genes required for this cell cycle transition and S-phase progression, such as those encoding the subunits of the helicase MINICHROMOSOME MAINTENANCE2-7 (MCM2-7) and the replication processivity factor proliferating cell nuclear antigen (PCNA) complexes (reviewed by Sabelli and Larkins, 2009c). RBR proteins are sequentially phosphorylated and inhibited by different CDK complexes, which relieves the block on E2F/DP-dependent gene expression and results in the transition into S-phase. In plants, CDK complexes containing D- and A-type cyclins seem to phosphorylate and thus inhibit RBR proteins. Grasses possess a more numerous and functionally diverse set of RBR proteins than most dicots, including *Arabidopsis thaliana* (Sabelli and Larkins, 2006, 2009c). The maize (*Zea mays*) genome contains at least four RBR genes that are grouped into two distinct types of duplicated genes, exemplified by *RBR1* and *RBR3* (Sabelli et al., 2005; Sabelli and Larkins, 2006). While *RBR1* functions as a repressor of cell cycle progression (Sabelli et al., 2013), *RBR3* stimulates the expression of genes encoding MCM2–7 proteins and DNA replication (Sabelli et al., 2009). *RBR3* is itself an E2F/DP target whose expression is negatively regulated by *RBR1* (Sabelli et al., 2005).

### DNA REPLICATION INITIATION FACTORS

Regulation of DNA replication in plants is generally believed to follow conserved eukaryotic patterns (reviewed by Costas et al., 2011). Initiation of S-phase requires priming of chromatin via the assembly, at origins of replication, of the pre-replication complex consisting of ORIGIN OF REPLICATION COMPLEX (ORC1-6), CDC6, CDT1, and MCM2-7 proteins. ORC1-6 associate with origins of replication throughout the cell cycle but become sequentially bound by different proteins. Interaction of CDC6 and CDT1 with ORCs during G1 promotes the loading of MCM2-7, effectively licensing the origins for DNA replication (reviewed by Tuteja et al., 2011). These replication origin complexes are activated by CDKs at the G1/S transition, which leads to the recruitment of the replication machinery, the unwinding of DNA through MCM-dependent helicase activity, and DNA synthesis with the formation of replication forks. MCM protein complexes are displaced from replication origins as replication forks advance along the DNA, which prevents re-licensing of origins until S-phase and M-phase are completed. In endoreduplication cycles, DNA replication is initiated independently of M-phase, resulting in repeated rounds of DNA synthesis in the absence of mitosis. However, endoreduplication results from specific cell cycle modifications rather than merely uncontrolled activation of replication origins, which would cause over- and incomplete DNA replication (Costas et al., 2011; De Veylder et al., 2011; Sabelli, 2012a).

### UBIQUITIN-DEPENDENT PROTEOLYSIS

Ubiquitin-Proteasome System-mediated proteolysis promotes the controlled destruction of several cell cycle regulators, which is critical for cell cycle phase transitions. Cyclins, CKIs, and other cell cycle regulators are targeted to the proteasome via their selective modification by various ubiquitin-protein ligases. Among the multimeric E3 ubiquitin-protein ligases functioning in plant cell cycle control are the anaphase promoting complex/cyclosome (APC/C), the Skp1/Cullin/F-box complex, and the Cullin-RING Ubiquitin Ligases (Heyman and De Veylder, 2012; Genschik et al., 2013). During late mitosis and most of G1, CDK activity is typically reduced via the targeting of A- and B-type cyclins to the proteasome by the APC/C. In addition to its roles in the cell division cycle, E3 ligases control endoreduplication cell cycles, those occurring in trichome and root nodule cells being perhaps the best characterized such examples (Cebolla et al., 1999; Roodbarkelari et al., 2010; Heyman and De Veylder, 2012; Genschik et al., 2013).

### CDK-SPECIFIC INHIBITORS

Non-catalytic inhibitors of CDK/cyclin complex activity, generally termed CKIs, have been identified as chief cell cycle regulators in all eukaryotes, and act by obstructing substrate interaction and ATP binding. Two types of CKIs have been identified and characterized in plants: INHIBITOR OF CDC2 KINASE/KIP-RELATED (ICK/KRP) and SIAMESE/SIAMESE-RELATED (Yi et al., 2014). ICK/KRPs typically bind to complexes containing A-type CDKs and D-type cyclins (Wang et al., 2008), although interaction with B-type CDKs was also reported (Nakai et al., 2006). Plant CKIs are targeted by several E3 ligases for degradation (Zhou et al., 2003; Weinl et al., 2005; Jakoby et al., 2006; Ren et al., 2008; Roodbarkelari et al., 2010). In mitotic cell cycles, ICKs/KRPs are phosphorylated by B1-type CDKs and targeted for UPS-mediated degradation, with consequent stimulation of A-type CDK activity and M-phase (Verkest et al., 2005).

The core molecular factors controlling the cell cycle in plants conform, to a large extent, to those identified in other higher eukaryotes. However, plant genomes are often characterized by a larger complement of key cell cycle genes as well as by uniquely possessing certain types of CDKs, E2Fs, and CKIs. The resulting complexity in cell cycle regulatory mechanisms, which appears to have considerable redundancy, may have evolved largely to fine-tune the cell cycle for the requirements of the sessile life style of plants.

## ROLES OF DIFFERENT CELL CYCLES AND REGULATORS IN SEED DEVELOPMENT

### EMBRYO

#### Cell proliferation, patterning, and morphogenesis during embryo development

From the first zygotic division through the early globular (8–16 cells) stages, many aspects of embryo development in monocots and dicots are conserved (Lau et al., 2012; Sabelli, 2012b). The zygote divides asymmetrically and generates an apical cell with dense cytoplasm and a large vacuolated basal cell at the chalazal and micropylar ends of the embryo sac, respectively, establishing early embryo polarity and patterning. The apical and basal cells produce, respectively, the proembryo and the suspensor, which has an embryo proper-nourishing function. Afterwards, cell proliferation and differentiation occur coordinately to produce all embryo cell types and tissues, including the cotyledon(s), which in dicots accumulate storage compounds and typically occupy a large fraction of the mature seed volume. In dicots, periclinal divisions of the octant cells result in the globular embryo. Subsequently, the heart stage is reached with the characteristic emergence of two cotyledon primordia as opposite lateral extensions of the apical end. Next, cell proliferation and differentiation of the basal cell tier lead to the torpedo stage. As growth proceeds under mechanical constraints imposed by the ovule, the embryo increasingly assumes its typical curved shape. During development, monocot and dicot embryos display increasingly different morphologies. Patterns of cell division and cell lineages usually become less organized in monocot embryos and while two prominent cotyledons emerge in dicots, the origin of the single monocot cotyledon, the scutellum, is spatially more variable. In dicots, cotyledon cells undergo endoreduplication (Dhillon and Miksche, 1983; Lemontey et al., 2000; Rewers and Sliwinska, 2012).

The importance of cell cycle control during embryogenesis extends beyond its most recognizable aspects related to cell division and endoreduplication cycles. In *Arabidopsis thaliana* and *Nicotiana tabacum*, disruption of proper developmental patterns through lengthening or impairment of cell division by interfering with CDKA;1 (Hemerly et al., 2000), DNA polymerase 𝜀 (Jenik et al., 2005), and A3-type cyclin (Yu et al., 2003) indicates the dependence of embryo patterning and morphogenesis on the correct execution of cell divisions. Also, loss-of-function of *HOBBIT (HBT)*, which encodes a homolog of the APC/C subunit CDC27 (Blilou et al., 2002), causes defects in hypophyseal cell specification and basal embryo cell division, perturbing root meristem formation (Willemsen et al., 1998). Recent investigation on *Arabidopsis* post-embryonic development indicates that moderate and high levels of CDKA;1 activity determine whether cells divide symmetrically or asymmetrically, respectively, and that CDKA;1 activity is conveyed via RBR1 and its control over cell cycle- and differentiation-related genes (Weimer et al., 2012). Similar mechanisms connecting CDKs and RBR1 appear to operate during embryogenesis (Nowack et al., 2012), and several studies have shed light on the roles of the RBR/E2F pathway, CDKs, D-type cyclins, CKIs, as well as additional APC/C components, in developing *Arabidopsis* seeds, underscoring the importance of precise cell cycle control for embryonic patterning, cell proliferation, and endoreduplication.

#### The role of D-type cyclins in embryogenesis

Collins et al. (2012) carried out a comprehensive investigation of D-type cyclin expression and function in developing *Arabidopsis* seeds. Various D-type cyclin genes show distinct and overlapping tissue-specific expression patterns during seed development. Developmental progression characterized in loss-of-function mutants revealed that embryo development is slower in the triple D3-type cyclin mutant, but not in single and double mutant combinations, indicating that this cyclin subtype is necessary for normal development, with individual, partly redundant components. Ectopic CYCD3;1 expression delays progression of embryonic development and causes atypical divisions in the hypophysis and suspensor. In contrast to CYCD3;1, ectopic expression of CYCD7;1, a previously uncharacterized cyclin that is not expressed in wild-type seeds, induces cell proliferation and cell enlargement in the embryo (and endosperm), causing excessive growth and higher seed lethality. These results suggest that adequate control of spatiotemporal patterns of cell division, through the regulation of specific D-type cyclins and thus possibly their CDK complexes, is important for embryo patterning and growth.

#### Roles of CDKs, the RBR pathway, and CKIs in embryogenesis

A combinatorial analysis of mutants recently allowed functional dissection of five members of the ICK/KRP-type of CKIs during *Arabidopsis* seed development and revealed a link between ICK/KRPs, the RBR/E2F pathway and cell proliferation (Cheng et al., 2013). CDK activity gradually increased as individual ICK/KRP T-DNA insertion mutants were combined, indicating that ICK/KRPs act at least partially as dosage-dependent CDK inhibitors. Although single-gene mutants and most multiple-gene mutants have wild-type morphological phenotypes, the quadruple *ick1ick2ick6ick7*, and the quintuple *ick1ick2ick5ick6ick7* mutants have a slightly altered leaf shape, suggesting some degree of redundancy among individual genes. The quintuple mutant has larger cotyledons, leaves, petals, and seeds than wild type. The ICK/KRP mutants generally have more numerous but smaller cells in all organs examined, and this phenotype is enhanced as the number of combined mutant genes is increased. The quintuple mutant displays extensive up-regulation of the E2F pathway via increased phosphorylation of RBR1, consistent with reduced inhibition of CDK complexes.

An additional connection between CDKs and RBR1 was provided by combinatorial analyses of their corresponding mutants (Nowack et al., 2012). Delayed development and drastically altered cell numbers and sizes are observed during embryogenesis in null *cdka;1* mutants (indicating that embryogenesis can adjust to variations of cell number and size), while loss of function of both CDKA;1 and B1-type CDKs leads to embryogenesis arrest. Because post-embryonic defects in *cdka;1* mutants can be restored by *rbr1* mutations (Nowack et al., 2012), CDKA;1 most likely acts via the RBR/E2F pathway to control embryogenic cell proliferation. *Arabidopsis DEL1* encodes an E2F-DP-like DNA binding protein that was previously shown to be mostly expressed in dividing cells and to inhibit endoreduplication (Vlieghe et al., 2005). A loss-of-function *del1* mutant exhibits a small (∼11%) but significant increase in seed size (Van Daele et al., 2012), although it was not determined whether this phenotype is due to stimulated endoreduplication. Collectively, these results suggest complex interactions among plant ICK/KRPs, which can function redundantly, but also in a dosage-dependent manner, to control the activity of CDK complexes, the RBR1/E2F pathway and cell proliferation.

#### The influence of the APC/C in embryogenesis

Functional characterization of genes encoding APC/C subunits and activators during embryogenesis revealed roles in cell-type specification and morphogenesis, in addition to cell division and endoreduplication. Mutations in both APC4 (Wang et al., 2012) and APC1 subunits (Wang et al., 2013) cause defective gametogenesis and developmental arrest during embryogenesis, which seem to be associated with accumulation of B-type cyclin and altered auxin distribution. *SAMBA* encodes a conserved plant-specific protein that binds to and potentially regulates the APC/C (Eloy et al., 2012). *SAMBA* expression is high during embryogenesis and, to a lesser extent, early post-embryonic development. Loss-of-function *samba* mutants have defective male gametogenesis and enlarged shoot and root apical meristems that result in the production of larger seeds, embryos, leaves, and roots. Increased organ size in *samba* mutants could be attributed to a larger number of more highly endoreduplicated cells, rather than to larger cells. SAMBA binds an A2-type cyclin, which is stabilized in *samba* mutants during early development. Thus, SAMBA negatively regulates cell proliferation at least partially by targeting A2-type cyclins for proteolysis via the APC/C.

In conclusion, precise cell cycle regulation, both spatially and temporally, is critical for embryogenesis and plant reproduction. The core cell cycle regulators CDKs, cyclins, RBR1, ICK/KRPs, and the APC/C seem to play concerted roles and thus affect asymmetrical cell divisions, cell proliferation and endoreduplication during embryogenesis.

### ENDOSPERM

#### Patterns of endosperm development

The endosperm functions in nourishing the sporophyte during embryogenesis and controlling germination and, mostly limited to monocots, also during early post-embryonic development. However, it also plays important roles in other aspects of seed development, including epigenetic regulation, coordination of cell patterning and proliferation, signaling among the major seed structures and control of seed size (Berger et al., 2006; Sabelli and Larkins, 2009b; Nowack et al., 2010; Fiume and Fletcher, 2012; Costa et al., 2014). The different cell cycle types occurring during the development of the persistent endosperm in grasses have mostly been investigated in traditional biological models and valued crop species, such as maize and rice (*Oryza sativa*).

**FIGURE 3:**
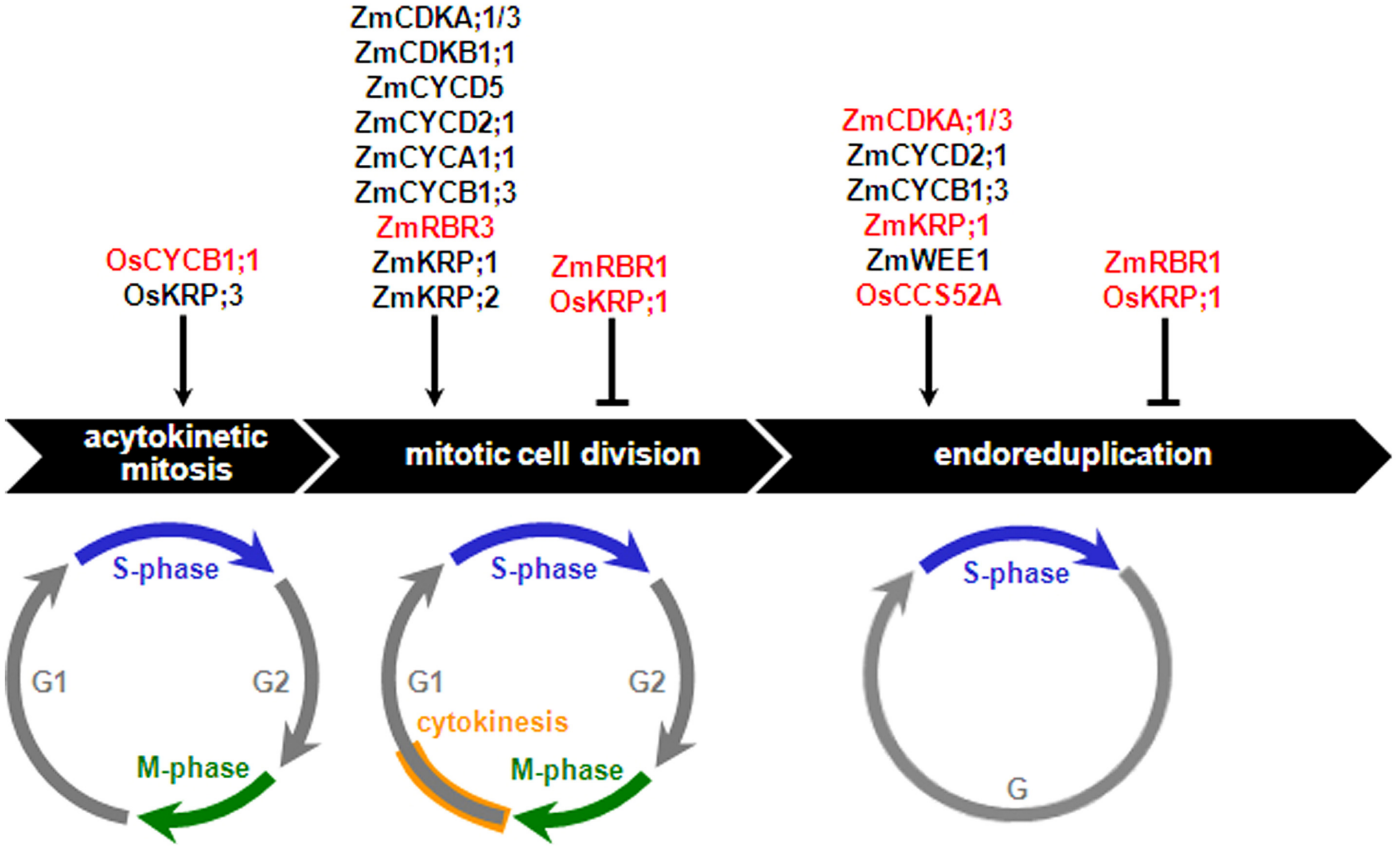
**Effects of core cell cycle regulators on the cereal endosperm cell cycles.** Core cell cycle regulators that positively or negatively regulate proliferative (acytokinetic mitosis and mitotic cell division) and endoreduplication cycles are shown. Net stimulatory or inhibitory effects are assigned as a function of cell proliferation- and ploidy-related phenotypes observed in mutant or transgenic analyses carried out either in planta or plant cell cultures (red-colored fonts) or inferred from a combination of expression analyses and biochemical assays (black-colored fonts), as discussed in the text. Os, *Oryza sativa*; Zm, *Zea mays*.

The nuclear type of endosperm development is the most frequently encountered pattern among Angiosperms, and with regard to its early stages up to cellularization, is highly conserved in monocots and dicots (Olsen, 2004; Sabelli and Larkins, 2009b; Becraft and Gutierrez-Marcos, 2012). In this developmental type, the primary endosperm nucleus and its derivatives undergo acytokinetic mitosis iteratively, resulting in a syncytium that can comprise up to thousands of nuclei that are initially distributed around the central vacuole of the central cell (Olsen, 2004). Anticlinal cell wall deposition, forming alveoli encasing individual nuclei, creates the first endosperm cell layer, and reiteration of anticlinal cell wall deposition, alveolation, periclinal cell wall formation and periclinal cell division results in centripetal generation of additional cell layers, gradually replacing the space occupied by the central vacuole with cells. Cellularization is typically completed within three to six days after pollination (DAP) in cereals and by the torpedo stage in *Arabidopsis*. In the following developmental stage, mitotic divisions coupled to cytokinesis result in cell proliferation, thus producing most of the endosperm cells. Past the cell proliferation stage, in nonendospermic species, such as *Arabidopsis*, the endosperm is absorbed by the rapidly developing embryo and is limited to a single or few cell layers at seed maturity. In contrast, in endospermic species, the endosperm is persistent and its development progresses through relatively conserved stages, comprising a period of cell enlargement typically associated with endoreduplication, followed by maturation involving programmed cell death (PCD) of specific cell types, dehydration, and dormancy. The roles played by core cell cycle regulators in the different cell cycles of cereal endosperm development are discussed in the following sections and summarized in **Figure 3**.

#### Maize endosperm development: an overview

Endosperm development in maize (and related cereals) follows the general monocot developmental pattern described earlier (Sabelli and Larkins, 2009b). Starting around three DAP, as the syncytium is cellularized, endosperm growth is mostly attained by an increase in cell number through mitotic cell divisions, which peak at eight to 10 DAP (Kiesselbach, 1949; Kowles and Phillips, 1985; Lur and Setter, 1993). Initiating in the endosperm central regions, also around eight to 10 DAP and then extending centrifugally, cells gradually and asynchronously cease mitotic cell divisions and switch to endoreduplication. As a result, the nuclei of many central endosperm cells reach high DNA content levels (some in excess of 200C; C = DNA content of haploid nuclei) and contain multiple, apparently uniform, copies of chromosomes (Bauer and Birchler, 2006). In agreement with the high correlation between ploidy level and cell size observed in numerous cell types and organisms, the spatiotemporal pattern of mitosis-to-endoreduplication switch in the maize endosperm creates a gradient of nuclear ploidy and cell size. Small non- or under-endoreduplicated cells are located mostly at the peripheral aleurone and sub-aleurone layers, as opposed to the increasingly large and endoreduplicated inner starchy endosperm cells. By 16 DAP, the expanded endoreduplicated cells account for most of the endosperm volume (Vilhar et al., 2002) and as many as 75% of its cells can become endoreduplicated at later developmental stages (Dilkes et al., 2002). Starchy endosperm cells typically display concomitant accumulation of starch and proteins with endoreduplication, which has long suggested a causal relationship between these processes (Larkins et al., 2001; Kowles, 2009; Sabelli and Larkins, 2009a,b; Sabelli, 2012b). Starchy endosperm cells subsequently undergo PCD (reviewed by Young and Gallie, 2000; Sabelli, 2012a), and the peripheral aleurone layer persists as the only living tissue past seed desiccation.

#### The contrasting endosperm development in *Brachypodium distachyon* and other model cereals

In comparison with maize, rice, and other major cereals, initial analyses of seed development in the emerging grass model, *Brachypodium distachyon*, have revealed important differences with respect to cell cycle control and its possible relationship with storage compound accumulation (Guillon et al., 2012; Trafford et al., 2013). In contrast to its related species, barley (*Hordeum vulgare*), *Brachypodium* endosperm displays reduced cell proliferation and enlargement. Also, the expression of a B1-type CDK and an A3-type cyclin is reduced, in agreement with a withdrawal from a mitotic cell cycle program, but >6C nuclei are absent, indicating no occurrence of endoreduplication. Along with these differences, *Brachypodium* endosperm cells exhibits limited starch deposition and thickened cell walls, the cell wall polysaccharide, β-glucan, representing the main seed storage carbohydrate. Accordingly, the expression of genes involved with starch biosynthesis is reduced. Thus, an interpretation of the contrasting endosperm developmental patterns in *Brachypodium* and other cereals is that starch accumulation drives endosperm cell enlargement (Trafford et al., 2013) and, consequently, high nuclear ploidy levels are correlated with large cell sizes.

#### The importance of early nuclear and cell proliferation activities for endosperm development

Regulation of the syncytium-to-cellularization transition seems to be key for endosperm development and seed growth. Rice *THOUSAND-GRAIN WEIGHT 6* (*TGW6*), which encodes an indole-3-acetic acid (IAA)-glucose hydrolase, appears to stimulate, by elevating IAA levels, the expression of CYCB2;2 and E2F1 during the first three days after fertilization; it also stimulates premature cellularization of the syncytium and reduces endosperm final cell number, grain length, and weight (Ishimaru et al., 2013). In addition, suppressing the expression of rice CYCB1;1 results in delayed endosperm cellularization and seeds containing only an enlarged embryo at maturity (Guo et al., 2010). Heat stress affects rice endosperm cellularization by interfering with the expression of the epigenetic regulator FERTILIZATION-INDEPENDENT ENDOSPERM 1 of the Polycomb Repressive Complex 2 (PRC2), thus reducing seed size (Folsom et al., 2014). In *Arabidopsis*, over-expression of *SHORT HYPOCOTYL UNDER BLUE 1* (*SHB1*) promotes early endosperm nuclear proliferation, a delay in cellularization, enlarged chalazal endosperm, and enhanced proliferation and expansion of embryo cells, leading consequently to increased seed size (Zhou et al., 2009). It is possible that these effects are mediated by members of the HAIKU pathway, which function downstream of *SHB1* to promote syncytial endosperm and seed growth via epigenetic control of cytokinin signaling (Li et al., 2013).

The rate and duration of proliferative cell cycles seem to also play an important role in maize seed size. Recently, Sekhon et al. (2014) examined transcriptional and developmental changes during seed development of maize populations that were selected for large and small seed sizes (termed KLS30 and KSS30, respectively). KLS30 seeds are more than fourfold heavier and twofold larger than KSS30 seeds and contain proportionally larger endosperms. Metabolite and genome-wide expression analyses indicated that, compared to KLS30 seeds, the linear phase of the grain filling (phase II) in KSS30 seeds initiates earlier, but it also occurs at slower rates and terminates earlier. Notably, KLS30 endosperms, relative to their KSS30 counterparts, display up-regulated sucrose metabolism and expression of the cell wall invertase INCW2 (encoded by the *MINIATURE 1* gene), which is required for normal endosperm cell proliferation and expansion (Vilhar et al., 2002; Chourey et al., 2006). KLS30 endosperms also exhibit higher expression at 12–18 DAP of several genes encoding D- and B-type cyclins and APC/C subunits. Although cell number, size, and nuclear ploidy were not determined in KLS30 and KSS30 endosperms, these gene expression profiles suggest higher mitotic activity in the former. The balance between cell proliferation and endoreduplication activities as a factor influencing endosperm and seed sizes is also supported by the analyses of multiple small-seeded popcorn inbred lines in comparison to typically large-seeded dent inbred lines (Dilkes et al., 2002; Coelho et al., 2007). Popcorn lines revealed higher endosperm ploidy levels from as early as 13 DAP in comparison to dent inbred lines. This difference could be attributed to an earlier transition between the cell division to endoreduplication stages, and/or higher rates of endoreduplication in popcorn lines. The importance of correct timing for cessation of cell proliferation and commencement of cell enlargement as an underlying factor for growth has been documented (reviewed by Powell and Lenhard, 2012). Collectively, these studies suggest a causal relationship between increased periods and/or rates of cell proliferation with larger endosperms (and, consequently, larger seeds), by establishing a stronger-sink tissue, one possessing more numerous cells that subsequently enlarge and accumulate larger amounts of storage compounds.

### SEED COAT DEVELOPMENT, CELL CYCLE CONTROL, AND EPIGENETIC CONTROL OF SEED DEVELOPMENT

In most Angiosperms, the two ovule integuments that enclose the nucellus differentiate into the seed coat following fertilization, and develop through stages of cell division, cell elongation, differentiation, and PCD in coordination with embryo and endosperm development (Haughn and Chaudhury, 2005). Investigation of *Arabidopsis* seed coat development revealed the existence of complex communication and interaction between these seed structures and that their underlying cell proliferation and enlargement are major determinants of seed size (reviewed by Nowack et al., 2010). Impairing the elongation of integument cells via the *transparent testa glabra 2* (*ttg2*) mutation reduces endosperm and seed growth (Garcia et al., 2005). *megaintegumenta*/*auxin-response factor 2* (*mnt*/*arf2*) mutants exhibit more numerous integument cells and enlarged seeds and embryos compared to wild type (Schruff et al., 2006). Premature syncytium cellularization reduces endosperm growth and integument cell elongation in *haiku* (*iku*) mutants (Garcia et al., 2005), whereas delayed cellularization and extended endosperm cell proliferation is associated with integument cell elongation in the enlarged seeds of *apetala 2* (*ap2*) mutants (Ohto et al., 2009). The enhanced cell proliferation in *mnt*/*arf2* and *ap2* mutants seems to be associated with increased expression of D3- and B1-type cyclins (Schruff et al., 2006; Ohto et al., 2009), indicating that the corresponding transcription factors repress cell divisions through a pathway that involves down-regulation of these core cell cycle regulators.

Epigenetic mechanisms are particularly important for integrating growth and development of the seed coat, embryo, and endosperm. Particularly among core cell cycle regulators, RBR1 is involved in gametophyte cell differentiation and endosperm nuclear proliferation along with epigenetic regulators such as PRC2 and DNA METHYLTRANSFERASE 1 (Ebel et al., 2004; Ingouff et al., 2006; Johnston et al., 2008; Jullien et al., 2008). *rbr1* mutants display fertilization-independent endosperm development, reduced cell proliferation in the ovule integuments prior to fertilization and impaired differentiation of the seed coat (Ebel et al., 2004; Ingouff et al., 2006).

In conclusion, there appears to be extensive crosstalk and coordination, in which epigenetic control and RBR1 play significant roles, between cell cycle activity in the developing seed coat and inner seed structures, such as the embryo and endosperm. These mechanisms could modify both signaling and mechanical constrains imposed by maternal tissues on developing seed structures, and consequently could control seed size (Haughn and Chaudhury, 2005).

## THE ROLE OF CORE CELL CYCLE REGULATORS IN THE CEREAL ENDOSPERM: THE MAIZE PROTOTYPE AND RELATED EXAMPLES

### CONTROL OF ENDOREDUPLICATION IN ENDOSPERM

The mechanisms that control the transition from the mitotic cell cycle into endoreduplication and its progression in various cell types and species have been recently reviewed in detail (Edgar et al., 2014; Sabelli, 2014). Among plant core cell cycle regulators, certain CDK/cyclin complexes, CKIs, APC/C activators and the RBR/E2F pathway have been functionally linked to the onset and/or rates of endoreduplication cycles. Although these cell cycle regulators are widely conserved across higher eukaryotes and appear to be recurrently deployed to produce cell cycle modifications that result in endoreduplication, their individual contributions may be species- and cell-type-specific (Roodbarkelari et al., 2010; Edgar et al., 2014). In maize endosperm, induced S-phase CDK activity and inhibited M-phase CDK activity were proposed to cause endoreduplication cycles (Grafi and Larkins, 1995). In support of this model, endoreduplication is inhibited and stimulated, respectively, by over-expression of a catalytically inactive, dominant-negative form of CDKA;1 (Leiva-Neto et al., 2004) and by decreased RBR1 activity and consequent up-regulation of E2F/DP-dependent gene expression (Sabelli et al., 2013). In addition, developing maize endosperm exhibits the contrasting expression of different CKIs (Coelho et al., 2005) and functionally distinct RBR homologs of the RBR1 and RBR3 types (Grafi et al., 1996; Sabelli et al., 2005, 2009, 2013). Endosperm endoreduplication particularly correlates with the potential inhibitory phosphorylation of CDK subunits by a WEE1 homolog (Sun et al., 1999a), differential cyclin expression (Sun et al., 1999b; Dante et al., 2014) and apparent down-regulation of UPS-mediated proteolysis of members of various cyclin types, including potential mitotic cyclins (Dante et al., 2014).

#### CKIs

ICK/KRP-type CKIs appear to have variable roles in different cell cycle types during cereal endosperm development. KRP;1 is expressed at nearly constant levels in 7–21 DAP endosperm, while KRP;2 protein levels decline during this period, suggesting a more positive role in endoreduplicating cells for KRP;1 than KRP;2, which in contrast could be preferentially involved with regulation of the mitotic cell cycle or its transition into the endoreduplication cycle (Coelho et al., 2005). Biochemical assays showed KRP;1 activity corresponds partly to a CDK inhibitory activity existing in endoreduplicating endosperm (Grafi and Larkins, 1995). KRP;1 and KRP;2 are able to partially inhibit the complex CDK fraction that binds p13*^suc1^*, and they specifically inhibit the CDK activity associated with A1- and D5-type cyclins, but not that associated with CYCB1;3 (Coelho et al., 2005). Overexpression of KRP;1 along with the wheat dwarf virus RepA protein, which antagonizes RBR1 (Grafi et al., 1996; Xie et al., 1996; Gordon-Kamm et al., 2002; Sabelli et al., 2005, 2009), causes ectopic endoreduplication in cultured, proliferating maize cells, indicating that coupling the stimulation of G1/S transition to the inhibition of certain CDK complexes is sufficient for endoreduplication onset in otherwise dividing cells (Coelho et al., 2005).

Expression and functional analyses *in planta* revealed that KRPs impact rice endosperm development. KRP;1 RNA is preferentially expressed at the mitosis-to-endoreduplication transition in wild-type rice plants, and its over-expression results in decreased kernel weight and filling rate, in addition to perturbed production and lower ploidy levels of endosperm cells (Barrôco et al., 2006). In contrast, KRP;3 RNA is most highly expressed in the syncytial endosperm, but its level declines subsequently in the cellularized endosperm, suggesting a specific function in the syncytial cell cycle or during the transition to cellularization (Mizutani et al., 2010).

### THE CDK AND RBR PATHWAYS

Recently, Sabelli et al. (2013) showed that RBR1 controls multiple molecular and cellular aspects of maize endosperm development. *RBR1* down-regulation via RNAi in endosperm cells results in enhanced expression of *RBR3*-type, *MCM2–7*, and *PCNA* genes. Mitotic and endoreduplication cell cycles are both stimulated by the alleviated inhibition of RBR1 on the G1/S transition, which causes RBR1-RNAi endosperm to have 58% more cells and ∼70% more DNA than its wild type counterpart by 19 DAP. However, this creates a surprising reduction in cell and nuclear sizes, in spite of increased endoreduplication, thus ruling out a causal and direct relationship between these processes, at least in the specific context of RBR1 down-regulation. Larger cell numbers and higher ploidy levels together cause a 43% increase in DNA content in mature endosperm upon RBR1 down-regulation, although no measurably altered storage protein content or kernel weight (a proxy for starch accumulation) were observed. Genetic interaction analysis of RBR1 and CDKA;1 (Leiva-Neto et al., 2004), down-regulated individually or in combination, indicated that CDKA;1 requires RBR1 for controlling endoreduplication, but conversely RBR1 represses downstream target genes independently from CDKA;1. These observations suggest distinct RBR1 activities at controlling endoreduplication, in which CDKA;1 probably participates via its inhibitory phosphorylation of RBR1, and at repressing E2F-dependent gene expression in a CDKA;1-independent manner. RBR1 down-regulated endosperm exhibits levels of p13*^suc1^*-adsorbed CDK activity similar to those of wild-type endosperm even in the presence of dominant-negative CDKA;1, indicating that various CDK complexes and RBR1 are negatively and reciprocally regulated, and implying that CDKs other than CDKA;1 participate in cell cycle stimulation upon RBR1 down-regulation (Leiva-Neto et al., 2004; Sabelli et al., 2013; Dante et al., 2014). Thus, perturbing RBR1 function revealed its key roles in integrating various processes in maize endosperm, but this did not translate in altered seed size and weight, suggesting the presence of a higher order, homeostatic regulation of endosperm development (discussed in next section).

The stimulation of CDK activity in down-regulated RBR1 maize endosperm prompts the question of the identity of CDKs, besides CKDA;1, controlling cell division and endoreduplication cycles (Sabelli et al., 2013). The identification and expression of different CDKs expressed in maize endosperm were reported recently (Dante et al., 2014). Previously uncharacterized CDKs of the A-type and B1-types, termed, respectively, CDKA;3 and CDKB1;1, were found to be expressed in endosperm. Protein levels of A-type CDKs are nearly constant throughout endosperm development, whereas expression of CDKB1;1 becomes markedly reduced during the transition into the endoreduplication stage and is stimulated upon RBR1 down-regulation. These observations are in agreement with the role of A-type CDKs in both mitotic and endoreduplication cell cycles and that of B1-type CDKs specifically in the mitotic cell cycle established in other species. Similar expression patterns, cyclin binding properties, and maintenance of nearly wild-type levels of CDK activity upon combined down-regulation of RBR1 and CDKA;1 collectively indicate that CDKA;1 and CDKA;3 are partially redundant or function coordinately (Sabelli et al., 2013; Dante et al., 2014). RBR1 down-regulated endosperm possesses more numerous cells and also exhibits higher ploidy levels, indicating that both cell division and endoreduplication are stimulated in distinct spatiotemporal patterns (Sabelli et al., 2013). These results suggest some redundancy among A-type CDKs and a specialized role for CDKB1;1 in positively regulating cell division during maize endosperm development (Leiva-Neto et al., 2004; Sabelli et al., 2013; Dante et al., 2014). Consistent with this interpretation, *Arabidopsis* possesses a single A-type CDK, and its B1-type CDKs can drive progression through cell division, but not endoreduplication cycles, in the absence of CDKA;1 and RBR1 (Nowack et al., 2012).

### CYCLIN/CDK COMPLEXES

The spatiotemporal expression of A-, B-, and D-type cyclins and their associated kinase activities in developing maize endosperm were recently investigated (Dante et al., 2014). Two main transcript expression patterns are apparent, one characterized by rapidly declining RNA levels with the onset of endoreduplication (A- and B-type cyclins), and the other with nearly constant RNA levels throughout endosperm development (D-type cyclins). However, these patterns are not consistent with those at the protein level, as shown by a discrepancy between declining *CYCB1;3* RNA in endoreduplicating endosperm but sustained levels of the encoded protein. While CYCB1;3 and CYCD2;1 proteins are localized to both the cytoplasm and nucleus of cells throughout the endosperm, CYCD5 protein is localized solely in the cytoplasm of peripheral cell layers. CDK activity associated with CYCA1 is tightly associated with cell division, while CYCB1;3-, CYCD2;1-, and CYCD5-associated CDK activities are highest at the transition from cell division to endoreduplication. These patterns together suggest roles for CYCA1 and CYCD5 in the cell division cycle, while CYCB1;3 and CYCD2;1 could participate in both cell division and endoreduplication. In particular, the switch to an endoreduplication program is marked by a drastic reduction in kinase activity associated with CYCA1. A-, B-, and D-type cyclins are more resistant to proteasome-dependent degradation in endoreduplicating compared to mitotic endosperm, which potentially contributes to the sustained levels of proteins, particularly CYCB1;3, in endoreduplicating cells. Consequently, the mitosis-to-endoreduplication transition and the accompanying cell enlargement typical of starchy endosperm cells are possibly associated with cell cycle modifications created by reduced proteasome-dependent proteolysis of several types of cyclins and, potentially, that of additional core cell cycle regulators (Dante et al., 2014).

### UPS-MEDIATED PROTEOLYSIS

Functional and expression analyses in rice revealed roles for APC/C activators that seem in part distinct from those of their homologs in dicots. Reduced expression of CELL CYCLE SWITCH 52A (OsCCS52A), a homolog of the APC/C activating subunit, results in smaller seeds and, despite reduced nuclear and cell size of endosperm cells, only modest reduction in their ploidy levels (Su’udi et al., 2012b). Also, reduced expression of the related OsCCS52B protein negatively impacts seed and cell sizes, but has no impact on endoreduplication (Su’udi et al., 2012a). Thus, collectively, OsCCS52A and B seem to play rather minor roles in rice endosperm endoreduplication, but have important roles in controlling cell and seed sizes. Although plant CCS52 homologs are known to promote proteolysis of A- and B-type cyclins and endoreduplication (reviewed by Heyman and De Veylder, 2012), the targets and mechanisms by which OsCCS52A and B control cell and seed sizes remain unknown. Some unidentified cyclins are presumably targeted by OsCCS52A and B, but the apparent down-regulation of UPS-mediated cyclin degradation and its contribution to sustained CYCB1;3 expression in endoreduplicating maize endosperm cells (Dante et al., 2014) further suggests that CCS52 homologs have a more significant role in cyclin proteolysis in mitotic as opposed to endoreduplicating endosperm. Also, these observations are consistent with others made in dicot model species, underscoring that various plant cell cycle regulators are targeted to UPS-mediated degradation by E3 ubiquitin ligases in cell-type- and cell cycle-type-dependent manners (Roodbarkelari et al., 2010; Heyman and De Veylder, 2012). Thus, dissecting the specific roles of E3 ubiquitin ligases and the UPS at large in governing various aspects of endosperm development, including cell cycle control, merits further investigation.

Besides the apparently reduced UPS activity in endoreduplicating compared to mitotic endosperm, translational regulation may also be responsible, at least in part, for sustained CYCB1;3 protein levels despite drastically reduced amounts of its RNA, as cyclin expression is known to be regulated at this level. In addition, many levels of gene expression regulation operate extensively during seed development, as a comparative analysis of the developing maize seed transcriptome and proteome revealed large discrepancies between cognate RNA and protein levels (Walley et al., 2013). Possible underlying mechanisms include differential stability of RNA and protein pairs, transport of proteins between tissues and out-of-phase circadian accumulation of corresponding RNAs and proteins (Walley et al., 2013). Thus, the expression of CYCB1;3 and other core cell cycle regulators in different seed structures may be subject to complex regulation.

## WHAT IS THE ROLE OF ENDOREDUPLICATION? EVIDENCE FROM THE MAIZE ENDOSPERM

Proliferative cell cycles are ultimately responsible for establishing the number of cells in a tissue, organ or body and, together with cell enlargement, determine their overall size. Although increased tissue/organ/body size resulting from stimulated cell proliferation has been documented in plants, typically the proliferation and enlargement of cells are inversely correlated, as more numerous cells are compensated for at the tissue/organ level by reduced cell size, essentially resulting in no overall differences (reviewed by John and Qi, 2008; Powell and Lenhard, 2012; Sabelli, 2014). A long-standing debate persists that opposes the “cell-based” or “cellular theory” (whereby cell proliferation and enlargement drive tissue/organ/body growth in a cell-autonomous manner) and the “organismal theory” (cell proliferation and enlargement follow a higher-order, supra-cellular program; Beemster et al., 2006; John and Qi, 2008; Sabelli, 2014).

While the impact of cell proliferation on tissue/organ/body size can be easily appreciated, that of endoreduplication is more controversial. Endoreduplication displays remarkable coincidence with cell enlargement in numerous cell types associated with different specialized functions. In an emerging and unifying view, endoreduplication facilitates cell expansion, growth, and accompanies differentiation in multiple cell types in which the occurrence of cell division could impair their function (Edgar et al., 2014). Among plant cells, the cellular functional specializations and attributes commonly associated with cell enlargement and endoreduplication include ability for rapid cell elongation (e.g., hypocotyl cells), branched cell morphology (e.g., trichomes), nutrient storage (e.g., cotyledons and endosperm of seeds and pericarp of fleshy fruits), hosting endosymbiotic bacteria (e.g., nodule giant cells) and interaction with pathogens and parasites (e.g., giant cells in galls and feeding sites). Some functions of endoreduplication in plant cells are only beginning to be elucidated (reviewed by John and Qi, 2008; Chevalier et al., 2011, 2013; De Veylder et al., 2011; Edgar et al., 2014; Sabelli, 2014). Recently, the contribution of endoreduplication to cell morphogenesis and cell identity maintenance (Bramsiepe et al., 2010), as well as regulation of gene expression and karyoplasmic homeostasis (Bourdon et al., 2012) were investigated. Bramsiepe et al. (2010) reported that, upon targeted reduction of endoreduplication levels by modification of core cell cycle gene expression, *Arabidopsis* trichome number is reduced due to dedifferentiation and resumption of mitosis by these cells. Conversely, promoting endoreduplication causes restoration of trichome cell identity, which revealed a role for endoreduplication in determining trichome cell fate. In endoreduplicated cells of the tomato pericarp, the nuclear surface has extensive grooves that are filled with mitochondria, thus allowing fairly constant nuclear surface/volume ratios, suggesting the existence of high ATP demand by nuclear processes (Bourdon et al., 2012). Accordingly, rRNA and mRNA transcription in individual nuclei is positively correlated with ploidy levels.

In the terminally differentiated starchy cells of maize endosperm, endoreduplication can be viewed as a mechanism that supports increased gene expression and the enhanced metabolic activity associated with cell enlargement and massive accumulation of starch and storage protein (Larkins et al., 2001; Sabelli and Larkins, 2009a; Sabelli, 2014). However, modification of endosperm endoreduplication, by perturbing the function of core cell cycle regulators, has challenged some aspects of this paradigm (Leiva-Neto et al., 2004; Sabelli et al., 2013). Similar to maize kernels in which the expression of a dominant-negative CDKA;1 reduces endoreduplication (Leiva-Neto et al., 2004), the size, weight, and morphology of RBR1 down-regulated kernels are essentially identical to their wild-type counterparts (Sabelli et al., 2013). Thus, evidence from modification of maize endosperm endoreduplication suggests this cell cycle does not contribute to metabolic and growth processes, at least at a whole-tissue level.

Surprisingly, RBR1 down-regulation in endosperm also results in coordinated reduction in cell and nuclear sizes (Sabelli et al., 2013). This indicates a role for RBR1 in coupling DNA content to nuclear and cell sizes. Importantly, these results invoke the existence of a causal relationship between nuclear and cell sizes in endosperm cells, which is not affected by perturbing RBR1 function, in support of the general karyoplasmic ratio theory. In addition, the observation of a larger number of smaller cells in RBR1 down-regulated endosperm seems to agree with the organismal theory of development. Consequently, one interpretation of the effects of RBR1 down-regulation in maize endosperm is that suppression of RBR1 function leads to enhanced cell proliferation, which results in a larger number of cells that, nonetheless, undergo less pronounced enlargement imposed by supra-cellular control of tissue size. Nuclear sizes are adjusted to the sizes of respective cells, regardless of their higher ploidy levels, which are achieved via enhanced endoreduplication. In RBR1 down-regulated endosperm, both the uncoupling of ploidy levels from cell and nuclear size and the reduced storage protein gene expression per unit of nuclear DNA possibly arise from increased DNA methylation and chromatin condensation (Sabelli et al., 2013), in a fashion similar to *Arabidopsis* cotyledon cells (van Zanten et al., 2011). Importantly, additional chromatin produced by enhanced endoreduplication in RBR1 down-regulated endosperm seems to be less transcriptionally active (Sabelli et al., 2013), providing latent transcriptional capacity in case cell enlargement is resumed or continued. Consequently, at the individual cell level, endoreduplication in the endosperm could function in the adjustment of nuclear size to cell size (thus preserving the karyoplasmic ratio) through a process influencing chromatin condensation states and transcription. This view seems to be supported by evidence from other model systems (Wu et al., 2010; Bourdon et al., 2012).

A deeper understanding of the role endoreduplication plays in endosperm development may require genetic analyses of this cell cycle by perturbing individual genes other than those encoding core cell cycle regulators, whose possible functions at coupling endoreduplication to cellular outputs may produce confounding results. The existence of compensatory mechanisms mediating tissue homeostasis may also require approaches based on a suite of technologies such as transmission electron and fluorescence microscopy, immunohistochemistry, DNA and RNA *in situ* hybridization, *in vitro* tissue culture and flow cytometry/fluorescence-activated nuclear sorting to unmask cell-autonomous effects (Gruis et al., 2006; Bourdon et al., 2012).

## CONCLUDING REMARKS

Acytokinetic mitosis, symmetric and asymmetric cell division, and cell enlargement greatly impact seed growth and development. During embryogenesis, correct execution of cell division is required for patterning and morphogenesis. Both the rates of proliferative and endoreduplication cell cycles (the latter of which being typically integral to cell enlargement and differentiation in storage compartments) and the timing of the developmental transitions between these cell cycles influence the final size of seed structures and ultimately that of the whole seed. In *Arabidopsis*, a pivotal role of its only RBR member, RBR1, in asymmetric cell divisions, gametogenesis and embryogenesis has been revealed, while in the developing cereal endosperm the RBR1 homolog is central to the control of cell proliferation and endoreduplication cycles and nuclear and cell sizes, underscoring the importance of this family of core cell cycle regulators for plant reproduction and development. Differential expansion of families of key cell cycle genes in various plant species seems to allow the establishment of both functional redundancy and specialization, creating complex cell cycle regulatory networks. Genome-wide analyses and functional gene characterization studies have recently begun to reveal potentially important differences in cell cycle control between dicots and monocots. These differences are evident from genetic analyses of members of the RBR family, whose increased complexity in grass species can allow functional diversification, as exemplified by RBR1 and RBR3. The APC/C and cyclin proteolysis appear to play less prominent roles in the control of cereal endosperm endoreduplication than in dicot root nodules, trichomes, and pericarp. More investigation is needed to unravel the functions of different cell cycle types and their underlying regulatory pathways in endosperm growth, development, and function.

## Conflict of Interest Statement

The authors declare that the research was conducted in the absence of any commercial or financial relationships that could be construed as a potential conflict of interest.
